# In the real world, people prefer their last whisky when tasting options in a long sequence

**DOI:** 10.1371/journal.pone.0202732

**Published:** 2018-08-20

**Authors:** Adele Quigley-McBride, Gregory Franco, Daniel Bruce McLaren, Antonia Mantonakis, Maryanne Garry

**Affiliations:** 1 Victoria University of Wellington, Wellington, New Zealand; 2 Regional Wines, Wellington, New Zealand; 3 Brock University, St Catharines, Ontario, Canada; Middlesex University, UNITED KINGDOM

## Abstract

When people in laboratory studies sample products in a sequence, they tend to prefer options presented first and last. To what extent do these primacy and recency effects carry over to real-world settings where numerous sources of information determine preferences? To investigate this question, we coded archival data from 136 actual whisky tastings each featuring seven whiskies. We analyzed people’s ratings of whiskies featured at different serial positions in the tastings. We found a recency effect: people gave their highest rating to whiskies in the last position, and voted the last whisky as their favorite more frequently. This recency effect persisted when we controlled for the counter explanation that whiskies with higher alcohol content tended to occupy later serial positions. The recency effect also persisted when we controlled for the age of the whiskies. Taken together, our findings suggest that the order of presentation matters in real-world settings, closely resembling what happens in laboratory settings with longer sequences of options.

## Introduction

Choice is an everyday dilemma. Every day, we evaluate our options and decide what we prefer. But having clear and complete information when making each decision, along with plenty of time to weigh various options, is a luxury. Instead, we often draw on whatever information is immediately available, make a decision in an efficient manner, and move on to the next dilemma. What kinds of information do we draw on? The opinions of those around us, our accessible memories of similar decisions, or how attractive each of the options appears are but a few obvious sources of influential information—but some are more surprising. Take, for instance, the position of an option in a sequence. If the same option was positioned at the start, in the middle, or at the end of a sequence of options, this could change people’s decisions about how much they like that option. That is, where an option appears in the sequence will result in different decisions about its quality, and whether it is preferred over other options. Here, we examine order effects in a real-world whisky tasting. We aim to determine the extent to which the influence of order on preferences persists when there is little control over other aspects of the environment.

Numerous theories in social psychology and behavioral economics discuss decision-making in real-world contexts. Choices are a product of the environment surrounding the decision-maker, where many features of the situation can influence the choices made. Whether people are aware of the context-specific influences on their choices does not matter, because people can be influenced by all types of information regardless of how noticeable it is [1]. For example, as mentioned in the introductory paragraph, the order of the options is one factor that might influence people’s decision without their awareness [2]. Organizations, governments, and sales-persons can capitalize on what is known about choosing, and change people’s decisions if they know how and when aspects of the social environment will affect people [3]. For example, even slight changes in the language used to inquire about a decision can change the types of decisions people make, such as asking if people would demand, take, or ask for an object [4]. Furthermore, in the presence of others, people automatically alter their behavior and decisions to be more cooperative [5] and will try to avoid negative emotions, such as unhappiness resulting from social rejection, by agreeing with the majority [4].

People, groups, and organizations can use knowledge about environmental effects on decisions to *create* a situation with particular features to influence people’s choices in a particular way (*choice architecture*; [1]). The goal of a choice architect is to be mindful of the various influences on choice, and to adjust these factors in the real world to produce desirable choices and outcomes. Choice architecture techniques are seen frequently in everyday life [3]. The layout of a grocery store, advertisements for various insurance plans, or, indeed, which product people decide they prefer at a tasting are all situations that have been “engineered” with economic or behavioral outcomes in mind. So, research has demonstrated that people can be “nudged” to prefer certain options by various means in many circumstances, including simplifying difficult choices, requiring active rejection of a default option, or, as is the focus in the current paper, ordering the options in a particular way [1].

How do we know that the order options appear in is important for how those options are judged? In the literature, the preference for the first and last options for sequentially presented items is called a *serial position effect*. The evaluation of sequences leads to serial position effects for a large range of stimuli, such as when people try to remember words [6], or judge competitors [7,8]. Furthermore, this preference for the first or last options has been demonstrated with sequences of products in controlled, laboratory settings with fast foods, soft drinks, and jellybeans [9,10]. In perhaps the most surprising example, people who sampled five glasses of wine generally preferred the first or the last wine [2]. The twist? All the glasses contained the same wine poured from the same bottle. But, even still, the sequence context encouraged people to see differences between the glasses of wine. These results in particular suggest that something about the order in which options are presented leads to a preference for the first and last products, given that the presence of other information was controlled in this laboratory study. Taken together, the literature suggests that we prefer the first and last options from among a sequence—even if the “options” are exactly the same. *Primacy effects* (better memory, or preference for the first item in a sequence) and *recency effects* (more frequent responses, higher recall, or preference for the last item in a sequence) can be found in a large range of contexts [6], and can occur in controlled laboratory studies (such as the 2009 study by Mantonakis and colleagues [2]) and more naturalistic field studies [11,12].

Why might the position of an option in a sequence change decisions about that option? One of the reasons that serial position effects occur is that a sequence thwarts a person’s ability to make absolute judgments, and instead encourages relative judgments [13]. For instance, if asked to decide how much we like a particular song when it is presented to us alone, we will tend to make an absolute judgment, a process that results in highly variable assessments between judges, because we will create our own comparison standard for the song [13]. But when an obvious comparison standard is available, we tend to make relative judgments whenever we can, such as when we are provided with two or more songs. That is, in a pair, people can make an opinion about which one is better than the other. Furthermore, people can easily rank longer sequences of products relative to one another in a similar way, which also reduces the variability in assessments seen for absolute judgments between people. But relative judgments can result in different evaluations of the same item if circumstances change. For example, people evaluate the same song better when it appears on a playlist of bad songs than great songs [13]. Why? Because when an option appears in a sequence rather than alone, people will judge that option by comparing it to the other options. In other words, any decision made about that option would be determined by how it sized up compared to the other options. Thus, relative judgments can lead to biased judgments in the context of a sequence.

There are circumstances that can reduce or eliminate the effect of serial position on judgments. In fact, for short sequences of fewer than four options, primacy effects are more common—people tend to prefer only the first option. Some research suggests that the first option is immediately put in the lead because, by default, it is the best so far as no other “competitor” has been presented [14,15]. Consider a wine tasting comprised of three different Pinot Noirs. As we try the first one, it becomes not only the lead, but it is also now a standard against which to compare subsequent options to. As we work our way through the other two options, we make relative judgments, comparing each one to the first option initially, unless another option beats it out earlier in the sequence [13]. Interestingly, other research suggests that the first option does not need to be “outstanding” as a competitor. Rather, the first option needs to merely meet a minimum threshold, or “satisfice”, in order to take the lead position initially [16]. We also tend to commit to a decision we have already made, which means that subsequent wines need to be noticeably better to compete with and replace the first wine [17]. In terms of probabilities, the current lead has a 70% chance of staying the favorite, but each challenger has only a 30% chance of becoming the new favorite [2]. Unless a subsequent option is exceptional, we are less likely to abandon a satisfactory first option. Taken together, research suggests that when people are faced with just a few options, the first one remains their favorite.

When sequence lengths are longer, the pattern changes, and two patterns of preferences tend to emerge. To see why, let us now consider a longer wine tasting, this one comprised of eight different Pinot Noirs. As with the smaller tasting, a satisfactory first wine is put in the lead and many people will stick with it [2,17]. But with longer sequences come more competitors that could potentially out rank the first wine. Because probabilities add up over the sequence, the more challengers the first wine faces, the more likely a new challenger will come out on top [2]. By the end of the tasting, therefore, people tend to favor the first wine or last wine. Moreover, people favor the other wines according to the position each one appears in the sequence, such that the least favored wine appears in the middle. Put another way, with longer sequences, people’s preferences form a U-shaped function.

Of course, outside of a laboratory, various other factors might change the “first and last” pattern demonstrated in controlled settings. Field studies examining memory for television commercials [18,19] and judgment of American Idol participants [12] are but a few examples that demonstrate the presence of typical serial position effects in more naturalistic settings. Other research suggests that serial position effects are different under some circumstances that would be found in the real world. For example, wine tastings draw people with a wide range of expertise [2]. Expertise about a product alters the approach people take when evaluating it, and altering that approach to evaluation can then change which option in a sequence people prefer [20]. Take, for instance, a very knowledgeable taster. This “expert” will decide on a favorite using the relative judgment process—pairing up each of the “challengers” in the sequence with the current favorite wine for comparison—and ultimately tend to prefer the first or last of the wines [2,13]. The main reason the expert adopts a strategy whereby each new “challenger” is compared to the current favorite is that experts are more excited by the products and motivated to evaluate them, so experts are more engaged and persistent when it comes to finding a competitor wine that is exceptional enough to demote the “champion” wine holding the lead [2].

A less knowledgeable wine taster will take a different approach. Because the novice wine taster is not well versed in the subtle differences among fine wines, he or she spends less time and effort comparing the “champion” wine to the other wines. A novice is incapable of the fine-grained pairwise comparisons that experts undertake, so less knowledgeable tasters assess each option, one at a time, on its individual merits—and isolated process of evaluation, which is more similar to an absolute judgment than a relative judgment [13,20]. So, rather than using the strategy of assessing whether this wine is better than the current favorite, a novice will simply try each one and evaluate it in isolation. Such an approach tends to result in a preference for the first option in the sequence because a wine novice rarely demotes the first wine, as the first one will “satisfice” their criteria [16]. The first option will remain the “champion” for novice tasters because the novices will lack the motivation to rigorously compare the first one to subsequent options in the way that an expert would [2].

Laboratory studies show that expertise is not the only factor that might influence serial position effects in the real world. In the context of a product sampling, such as a wine tasting, the host can also influence preference judgments. In some ways, a tasting host can be described as a “choice architect” who is creating a situation in which people will make decisions [1]. The host will have goals regarding the choices people make—does the host want people prefer a particular option, or are they seeking honest evaluations of the options? That is, any external guidance a person receives might change the effects of serial position and expertise [20]. One study sought to demonstrate why a host might influence the pattern of people’s preferences. In this study, the computer guided people through a tasting of five wines in a way that matched or did not match the way that people typically evaluate wines at their level of expertise (thereby encouraging or discouraging fine-grained, pairwise comparisons [20]). When the computer “host” was guiding the participants in a way that matched their expertise level, the decision process felt familiar, easy, and required little attention and time [21]. Familiar decision-making conditions discourage analytical decisions, and therefore encourage people to rely on heuristics to decide what they prefer. That is, people will rely on the evaluation strategies that they use most often because familiar circumstances result in fewer of the characteristics that tend to suggest the decision requires more careful thought. Therefore, when using a familiar evaluation strategy, wine experts tended to prefer the first and last options, and the wine novices tended to stick with the first options.

In contrast, when people were guided through the tasting in a way that did not match their level of expertise, the decision process felt unfamiliar—difficult to process and requiring more attention [21]. When people notice circumstances that may make decisions difficult, they will put more effort into decisions [21,22]. In the context of Philp and Mantonakis’ experiment, when the wine evaluation strategy that was encouraged by the “host” was unfamiliar, people of all levels of expertise became more analytical. Pushing people to switch to an analytical decision-making style diminished preferences for the first and last options in the wine tasting [20]. Taken together, laboratory research suggests that at a wine tasting, the guidance of a host could either inspire biases to choose the first and last products, or reduce them, depending on the direction the host takes the tasters, and the expertise of the tasters. Whether or not the host (“choice architect”) intends this outcome does not matter—the way they organize the tasting will influence order effects [1]. In addition, the results from the “computer-host” study add weight to the claim that expertise changes your evaluation strategy: Experts found a pairwise, fine-grained comparison strategy familiar, but novices did not [20]. Therefore, novices and experts can both contribute to order effects when sampling serially presented options, but only when both groups are left to use a preferred evaluation style.

Considered as a whole, laboratory research shows that when it comes to preferences, serial position matters for judgments of a variety of situations and stimuli. Furthermore, serial position effects have been demonstrated in naturalistic settings, when there could be a wide range of factors influencing preference for items that are presented in a particular order. In other words, serial position effects can shine through all other potential influences in the field. However, obtaining field data that has been collected and recorded in a largely systematic way in the real world is hard to come across. As discussed earlier, an ordinary product tasting would have social, individual, and commercial influences all at play [1,5], on top of the cognitive biases predicted by psychological research. Would people in an actual tasting of products prefer the first and last options, as laboratory research predicts, and how can we quantify these influences in the field? To address this question, we obtained and analyzed data from 136 whisky tastings, including a total of 952 whiskies, hosted by a liquor shop over the last decade and administered, for the most part, using an identical procedure at each tasting.

As we have already suggested, at a real tasting a multitude of information can change people’s preferences. This information includes, but is not limited to, memory for whiskies or flavors that people have enjoyed or disliked in the past (see the availability heuristic, [22]); social influence from others at the tasting when they voice their opinions [5,23]; expertise of the taster [2], and the influence of the host [1, 20]. Though we cannot control for all of these, as would be the case in a controlled laboratory study, we can control statistically for alcohol content, whisky age, the differences between each tasting and each whisky, and the number of people in attendance. We cannot control for influences that were not measured, of course, but we expect many such influences would not introduce systematic variance but instead random variance. Therefore, we would expect these unknown influences to randomly affect whiskies at various serial positions, thus creating noise in the preference data, or averaging out over the many different whisky tastings administered over the ten-year period.

If serial position matters in a real-world consumer setting, then we would expect to see tasters show preference for the first and last whiskies in the tastings compared to the whiskies presented in the middle. But to the extent that other sources of information weigh into tasters’ evaluations more than serial position, then we would expect no particular preferences based on the serial position of a whisky. These data are most useful in an applied sense—does a salesperson need to be mindful of sampling or presentation order when marketing their product? Can the salesperson alter a product-sampling situation in their favor by simply ordering the options in a particular way? Will this situational change be influential enough to “nudge” people towards a particular choice [1]? The literature suggests that the answer is “yes”, but these field data can tell us how much serial position can influence people’s judgments of products by drawing on information collected over a large period of time, using a fairly systematic procedure. These field data will allow us to infer the influence of presentation order over and above the factors we can account for in the analyses, and will also tell us whether serial position effects shine through despite the multitude of biases and influences that were unmeasured that could be affecting evaluations of each whisky product.

## Method

### Participants

We obtained archival data from 136 whisky tastings held between 2002 and 2013 at a liquor shop in Wellington, New Zealand, with a mean number of 29.73 (*SD =* 15.11) people at each tasting. This research was approved by the School of Psychology Human Ethics Committee (approval number: 0000020582) under delegated authority of Victoria University of Wellington. For statistical analyses, whiskies were treated as subjects, and the mean ratings the entire group gave each whisky at the tastings served as the dependent variable.

Because these were archival data, we had no control over the degree to which certain information about the attendees, whom we call "tasters," was in the data. After September 2011, some characteristics of the tasters were recorded, such as how many people identified themselves as an “enthusiast.” The definition of “enthusiast” varied between tastings, but it was most often defined as someone who had been to a number of tastings before, visited a distillery, or had been to a whisky festival. The number of people who had never been to a tasting before was also recorded (“first-timers”). In total, 20 tastings recorded a proportion of enthusiasts in attendance (*M =* 0.24, *SD =* 0.24) and 39 recorded a proportion of first-timers attending (*M* = 0.18, *SD* = 0.18). Unfortunately, we could not compare enthusiasts and first-timers due to lack of consistency in the definition of “enthusiast” between tastings, and the amount of missing data for these variables.

### Procedure

The following procedure describes a typical tasting session at the liquor shop during the years that we have coded and analyzed. Each tasting began with the host speaking about the area of the world (usually Scotland) from which the whiskies originate, how they are made, and each of the whiskies to be tasted (usually seven). The same host has hosted myriad tastings at this shop for the past decade, and hosted all the tastings in the dataset.

In a typical testing, each person received a “tasting sheet,” in which the seven whiskies were lined up in an unknown order with space underneath to write thoughts and scores. A standard whisky tasting pour is much less than a standard alcoholic drink size—typically around 20 to 22 milliliters, or 0.6 to 0.7 ounces. At the bottom of the tasting sheet, the whiskies were listed, and the prices on a separate sheet. One whisky remained a “mystery” though, and tasters were encouraged to attempt to name the whisky during the tasting. Therefore, although tasters knew which whiskies were at the tasting (except for the “mystery” whisky), they were completely blind to the order in which they tasted them. For example, they might have known that one of the seven whiskies is from the Lagavulin distillery, aged 16 years, but they did not know where in the sequence this whisky was featured. So, even if people knew that one of the whiskies that were present at the tasting was supposed to be objectively the best, they could not know which one it would be. As such, we were measuring people’s views about which whisky was the best in their *subjective* opinion, not how well people can tell which whisky is *objectively* superior. Usually, the host was also unaware of the order that the whiskies appeared in, as the manager of the shop determined this prior to the tasting. Sometimes the tasting was repeated on another evening though, and the order often remained the same for subsequent iterations of the same tasting.

The tastings had three phases. First, as a group, everyone smelled each whisky. On a whiteboard, the host recorded the words attendees called out to describe their experience as well as how many people selected each whisky as their favorite “nose.” Next, the group tasted each whisky, again contributing descriptors to be recorded on the whiteboard, and tasters voted for their favorite whisky based on taste. Between each whisky, the tasters drank beer and water as palate cleansers to remove the taste of the previous whisky. This phase lasted about 30 minutes, with 5 to 6 minutes dedicated to each whisky.

Finally, the session would finish with free time, during which tasters could try all the whiskies again as many times as they liked and in any order, to produce an overall score for each whisky, on a scale from 1 to 10. Although they used a 1 to 10 scale, tasters were told that 5 is a “pass,” and if they scored any whisky below 5, they had to give that whisky (a “fail”) away to someone who “passed it.” A 6 was considered a “good” whisky, a 7 is “very good,” an 8 is “excellent,” above 9 is “stunning,” and 10 is “perfect.” The host asked people to round their final scores to the nearest whole number. Counts were taken for each number, for each whisky, and recorded on the whiteboard. Then, a mean score for each whisky was computed based on these data, and written on the whiteboard. At the end, the host reveals which whisky was at each serial position, and the name of the “mystery” whisky.

For each of the 136 sessions spanning 11 years, the host photographed the whiteboard on which he recorded these data. We used these photos to extract information about the whiskies offered at each tasting, such as alcohol content, age, distillery, and name. If the information was not complete, we referred to the tasting sheet from that evening, and searched the Internet (for example, on whisky enthusiast websites) to fill in missing information. We also recorded the presentation order (serial position) of the whiskies.

For each session, we calculated the number of tasters by adding up the number of votes recorded on the whiteboard. The modal number of people was selected if there were inconsistencies, probably caused by someone who forgot to vote or recording inaccuracies. We also extracted data on the number of “experts” and “first timers” in attendance when this information was available. For each session, we also extracted data on the number of tasters who preferred the smell of each whisky (“favorite nose”), the taste of each whisky (“favorite taste”), and the final overall ratings for each whisky (“mean overall rating”).

The first author coded 100% of the whisky tastings. To check for accuracy, another research assistant coded serial position, alcohol content, “favorite nose”, “favorite taste”, and “mean overall rating” for 20% of all the whisky tasting sessions. We used Cohen’s kappa [24] to determine inter-rater agreement for this portion of the data, which was high (favorite nose, κ = .0.86, 95% CI [.85, .87], *p* < .01; favorite taste, κ = .0.82, 95% CI [.81, .0.84], *p* < .01; mean overall rating, κ = .0.81, 95% CI [.767, .0.86], *p* < .01). Disagreements were resolved by discussion. For example, there were a number of occasions where the writing on the whiteboards was very difficult to read, causing the discrepancy. Another example was that the whiteboard provided two types of data for “total number of tasters,” yet the two sources did not result in the same total number sometimes. In cases of discrepancy, we always adopted the more conservative resolution. For example, in the latter example, we recorded the lower number in the final data set.

## Results

Across 11 years and 136 whisky tastings, we obtained data for 952 whiskies. Of these, 98.7% were from Scotland; the remainder were from Japan and other locations in Europe. Their mean age (time spent in cask before bottling) was 16.07 years (*SD* = 7.09 years; *Mdn* = 15 years) with a range of 1 to 50. Mean alcohol content was 50.44% (*SD* = 6.67%, *Mdn* = 50%) with a range of 40% to 66.8%.

We now turn to our primary research question: across these 11 years, 136 whisky tastings, and 952 total whiskies, did the serial position of the whiskies matter? To answer this question, we treated the whiskies as subjects and the mean final rating for each whisky as the dependent variable. In addition, we were concerned that some of the other aspects of the tasting or procedure might masquerade as or mask sequence effects. For example, the manager at the whisky shop tended to place whiskies with higher alcohol content later in the tasting, at a higher serial position. Perhaps tasters simply preferred whiskies with higher alcohol, or more aged whisky? It is well known, even by novice tasters, that older whiskies and higher alcohol content whiskies taste different from those that are younger or less potent. Therefore, controlling for these aspects of the whiskies could help us to uncover “true” serial position effects, rather than effects caused by a confound. Although we could not account for all possible influences and sources of information that could affect preferences in a real tasting, we did have alcohol content and age information for the majority of the tastings and whiskies. So, our analyses controlled for the alcohol content and age of each whisky, and also allowed for the fact that the whiskies are nested within each tasting. That is, in each different tasting, slight differences—as well as the tasters who attend—all combine to contribute to mean overall ratings, and to the variation in these ratings. As a result, the variation in the allocation of preference and mean overall ratings attributable to each separate tasting and each separate whisky needed to be accounted for in our analyses as much as possible.

To address these issues, we used a crossed random effects multilevel model analysis, with tasting and whisky as two levels of variation within the model (216 different whisky types at 136 different tastings). There were two dependent measures of interest. The “favorite taste” variable provides valuable information here, because it is the closest measure of initial preference in this field study procedure, so these data and results are more similar to past laboratory studies. However, there was a large amount of missing data (22.1%) and the distribution was not normal (assessed using the Shapiro-Wilk test of normality, as well as viewing histograms and Q-Q plots to examine the distribution). Even though we used the LMER package in R, which addresses missing data concerns, these data violated some important assumptions of regression. The second dependent variable of interest is the “mean overall rating” measure. These data had lower levels of missing values, and were much closer to a normal distribution (assessed using the same methods, showing that these data were less skewed). However, because people were able to resample and change their scores as much as they wanted, this was less controlled in terms of the procedure and technique used to evaluate the whiskies. But, if both measures showed similar serial position effects, then we can be fairly certain that serial position effects are present in these data. The intraclass correlations (ICCs) for “favorite taste” of whiskies, and “overall mean rating” of whiskies indicated sufficient variation due to these higher level variables to warrant a multilevel model analysis for all variables except for the impact of tasting on “favorite taste” (ICC whisky, “favorite taste”: 0.26, 95% CI [0.17, 0.34]; ICC whisky, “overall mean rating”: 0.41, 95% CI [0.34, 0.47], and ICC tasting, “overall mean rating”: 0.10, 95% CI [0.05, 0.16]). Therefore, the model fitted for the “favorite taste” analysis did not include the tasting as a higher-level variable.

We accounted for the recorded variables that, theoretically and practically, could influence the ratings of whiskies: the tasting session, the specific whisky, the alcohol content and age of the whisky, and the number of people at the tasting. Even after accounting for these variables, the serial position of each whisky at a tasting mattered for both outcome measures. People chose the last whisky as their favorite more often than whiskies at other serial positions, and people rated the last whiskies higher than whiskies in other positions. The recency effect held even though other information, such as features of the whisky or tasting session, was included in the model to account for the effects of these potential confounds and real world circumstances. In other words, alcohol content, age of the whisky, and serial position were all significant predictors of which whisky people thought tasted the best and which whisky they rated the highest at each tasting—even when we accounted for the variance attributable to each whisky and each tasting.

**Fig 1:**
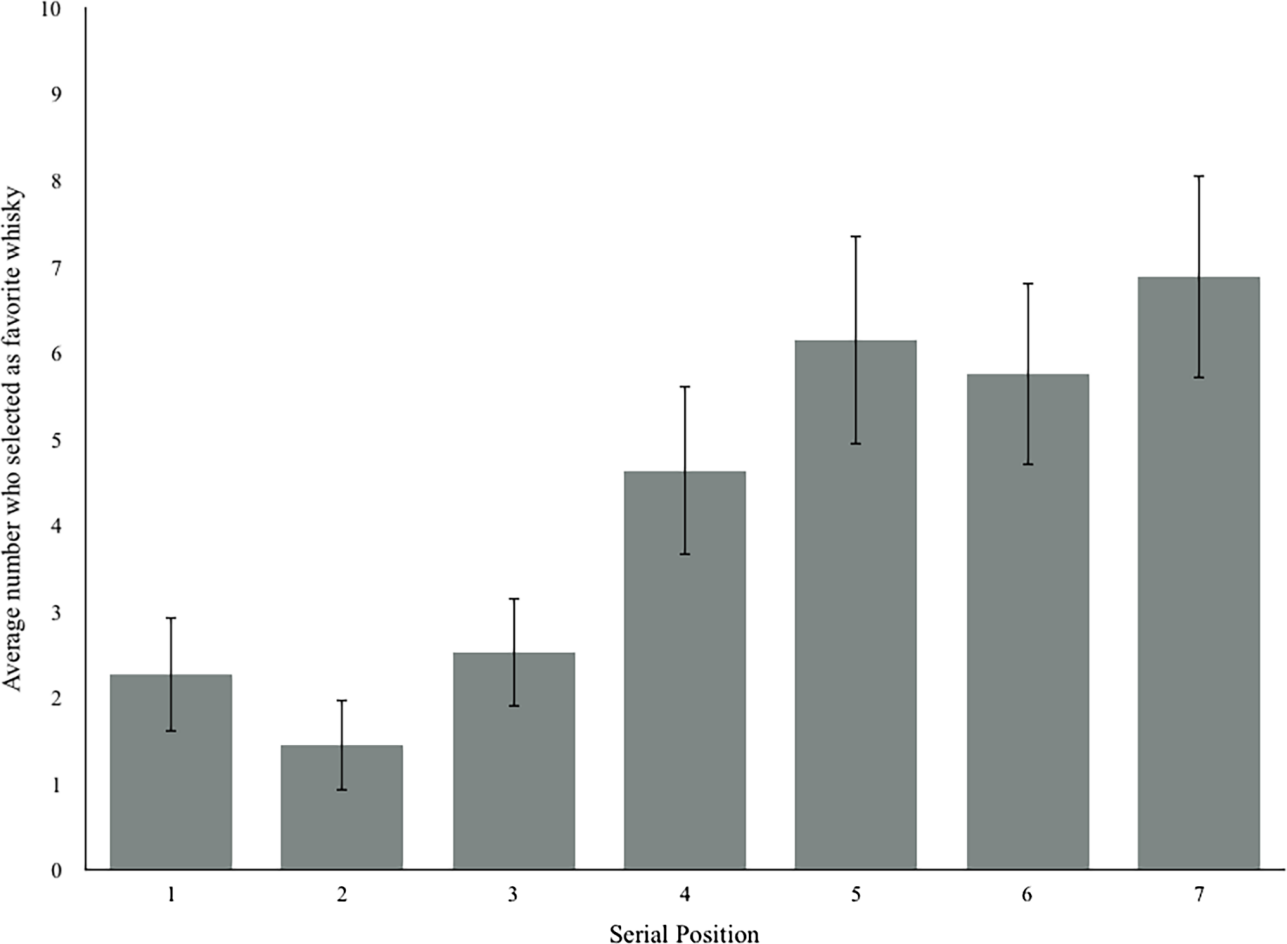
Mean number of people who selected whiskies in each serial position as their favorite after the first tasting. Error bars represent 95% confidence intervals of the mean.

More specifically, as Table 1 and Fig 1 show, people chose older whiskies (*B* = 0.09, *p* = 0.002) and higher alcohol content whiskies (*B* = 0.23, *p* < .001) as their favorite more often than younger whiskies with lower alcohol content. But also, people chose the last whisky as their favorite more frequently overall than whiskies at earlier serial positions (*B* = 0.64, *p* < .001). In addition, older whiskies (*B* = 0.01 *p* < .01), and higher-alcohol whiskies (*B* = 0.054, *p* < .01) were rated higher on average than younger or lower alcohol content whiskies (see Table 2). Furthermore, whiskies in later serial positions received higher ratings than whiskies in earlier serial positions (*B* = 0.10, *p* < .01).

**Table 1:** Summary of the relative impact of the predictors in the multilevel model with tasting and whisky as levels, and the number of people who chose each whisky as their favorite as the outcome variable.

Variable	*B*	*S*.*E*.	*t*	*p*
Intercept	-14.504	1.7672	-8.207	< .001
Alcohol content	0.2266	0.0339	6.693	< .001
Whisky age	0.0915	0.0296	3.092	0.002
Serial position	0.635	0.0941	6.749	< .001
Total number of judges	0.113	0.0133	8.525	< .001

*B* is the standardized regression coefficient for the multilevel mode, *S*.*E*. is the standard error of the standardized regression coefficient, *t* is the test statistic for the significance test of the predictor variable, *p* is the significance levels of the significance test of the predictor variable. All values are rounded to 4 dp.

**Table 2:** Summary of the relative impact of the predictors in the multilevel model with tasting and whisky as levels, and the mean overall rating of each whisky as the outcome variable.

Variable	*B*	*S*.*E*.	*t*	*p*
Intercept	4.699	0.2643	17.778	< .01
Alcohol content	0.0535	0.0051	10.870	< .01
Whisky age	0.0139	0.0041	3.365	< .01
Serial position	0.0997	0.0132	7.540	< .01
Total number of judges	0.0013	0.0022	0.574	0.5672

*B* is the standardized regression coefficient for the multilevel mode, *S*.*E*. is the standard error of the standardized regression coefficient, *t* is the test statistic for the significance test of the predictor variable, *p* is the significance levels of the significance test of the predictor variable. All values are rounded to 4 dp.

Together, the higher-order and predictor variables included in the “favorite taste” analysis accounted for 45% (*R*^*2*^_*c*_ = 0.4507) of the variation in “favorite” counts, with 32% (*R*^*2*^_*m*_ = 0.3281) of that due to including serial position, alcohol content, whisky age, and number people present in the analysis. Similarly, the full mean overall rating analysis accounted for 53% (*R*^*2*^_*c*_ = 0.5294) of the variation in preference scores, with 32% (*R*^*2*^_*m*_ = 0.3211) of that variation solely accounted for by serial position, alcohol content, whisky age, and number of tasters present. From this we can conclude that the variables included in our model explain a large portion of the variation in peoples’ ratings of the whiskies. When the model with “overall mean rating” was run without serial position to calculate the *R*^*2*^ change due to this variable alone, we found that 4% of the changes in people’s ratings were due to serial position alone. Alcohol content was also excluded from the analyses to calculate its *R*^*2*^ change for comparison. Alcohol content explained more of the variation in people’s ratings, with 18% explained by alcohol content of the whisky alone.

**Fig 2:**
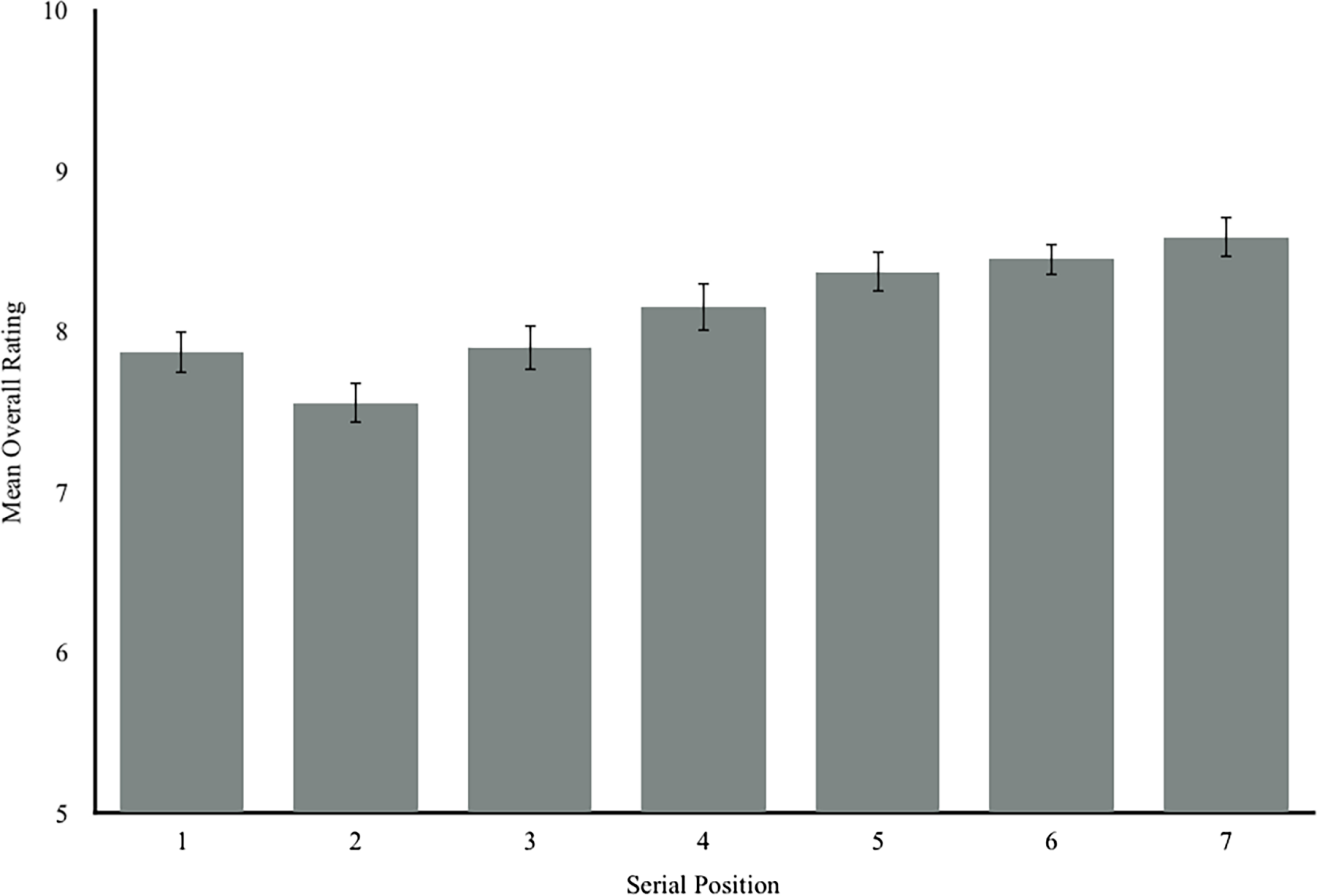
Mean overall rating given to whiskies in each serial position at tastings. Error bars represent 95% confidence intervals of the mean.

In short, serial position has a small impact on people’s preferences in the real world. Fig 1 shows the number of people who selected whiskies at each serial position as their favorite on average. Fig 2 shows the mean overall ratings for whiskies at each serial position and illustrates that tasters rated the whiskies in the last serial position higher overall. These figures also show that preference appears to steadily increase over the course of the tasting. For example, as serial position increased, mean rating increased, resulting in a mean difference of 0.43 between ratings of the last whisky (*M =* 8.59, *SD* = .74) and the middle whisky (*M =* 8.16, *SD* = .86)—a small but consistent increase in preference for later whiskies. Fig 2 also illustrates that, while the whiskies that were presented first appeared to be rated higher than the whiskies in the second position, the later serial positions (position 3 and beyond) quickly surpassed the first whisky.

## Discussion

Taken together, these findings suggest that when people face a sequence of whiskies with the task of deciding a favorite, the age of each whisky and how much alcohol it contains will both play a part in that decision. But the position in which a whisky appears will matter over and above its age and alcohol content. In addition, we found that recency effects persist in a real world tasting even though there are numerous influences that could result in people selecting whiskies in any serial position. So, although these results partly square with previous research on preferences [1,2], they do not in other ways. Our results suggest that tasters did not prefer whiskies that were presented first. How are we to explain this discrepancy with earlier work? How can we make sense of this preference for last options but not first options?

We suspect the answer is that all whisky tastings were long sequences of seven whiskies. We know that longer sequences (of four or more options) generally lead to a preference for last options, and reduced or no preference for first options [8,9,2]. In contrast, shorter sequences of less than four options tend to result in primacy effects more often [9,14]. For example, in one study, people tended to prefer the first option in short sequences of only two products, when they were in quick succession or more spaced out over time [14].

There is also another factor to consider: some people are more willing to change their minds than others; in the literature, they are said to have "low choice inertia" [25]. People with low choice inertia should tend to be more open to demoting a “champion,” and therefore tend to be unlikely to stick with the first option. They might also tend to prefer the last whisky at the tasting. We might expect that people at tastings would be relatively open to new experiences and tastes, but future research should investigate the extent to which attendees have a high degree of people with low choice inertia.

There is one obvious counterargument for the reason behind the observed preference for the last whisky seen in these tastings. It is reasonable to assume that by the time the last whisky is sampled, people are fairly inebriated and potentially less discriminating. However, the structure of the tasting is such that people are encouraged to go back and re-sample the rest of the whisky in each serial position and “fine tune” their scores before the formal tasting ends. Thus, re-sampling and fine-tuning occur when people are at the highest level of inebriation and perhaps a sub-optimal level of discrimination. As a result, there is no good basis for the idea that the preference for last is solely or even largely due to inebriation. In addition, the “inebriation hypothesis” would be applicable only to the “mean overall rating” variable, because the tasters would have consumed a fair amount of whisky by the time they are submitted their ratings. But although not as pristine, the analysis of “favorite taste”—a count of the number of people at each tasting who selected each whisky as their favorite as the outcome measure—was taken immediately after the tasters have had their first small sip of each whisky. Even so, we found the same patterns for “favorite taste” and the “mean overall score” variables (compare Fig 1 and Fig 2). That is, even when people used their first sip of each whisky to decide which was their favorite, the preference for the last whisky remained. Therefore, inebriation is unlikely to be the explanation for the consistent recency effect we found.

It is also important to note the obvious shortcomings of research using real-world data. One such shortcoming is that the procedure was not uniform between tastings, though it did adhere to a template. People could try the whiskies again after going through the tasting sequence, according to their own whims, and revise their scores. This real-world feature of the whisky tastings makes people's preferences for the last whisky all the more surprising. Now, we know that people tend to prefer the last option even when they are free to deliberate about the decision for longer, and are encouraged to be more analytical. Our findings therefore suggest that time pressure and heuristic processing styles [2,20,21] or the social environment [1] are only some of the reasons why people might prefer first and last options in a setting where they sample products.

Even so, with the variables that we assessed, we were explaining a large portion of the variation in preference scores with the variables that were available to us. In fact, approximately 32% of fluctuations in peoples’ preferences were explained by serial position, alcohol content, whisky age, and number of tasters present, and 53% when the higher level variables of the specific tasting and whisky were taken into account. Furthermore, approximately 4% of changes in people’s preferences were explained by serial position alone. We cannot identify all of the factors that might influence preference decisions in the real world, but these data do suggest that serial position plays a more important role than anticipated. It is reasonable to assume that other information, such as past experience with whisky, memory for other whiskies, and the preferences of others at the tasting, would also have had an impact [1,3]. Although this information could not be accounted for in our analyses, we can still attribute a moderate amount of variation in preference to order effects.

We have shown that the extent to which laboratory results for tasting a sequence of alcoholic beverages occur in a real-world setting. We were able to adjust for a large portion of the information available to the tasters that could influence their preferences—number of tasters, alcohol content, and age of the whisky could all be relevant to picking a favorite, though not linked to any particular glass of whisky at the tastings. But the recency-effect pattern persisted through the other factors we could not control for, that are common in everyday situations. These factors include, but are not limited to, memory for whiskies or flavors that people have enjoyed or disliked in the past (see the availability heuristic, [22]); social influence from others at the tasting when they voice their opinions [23], and the influence of the host [20]. Ultimately, our data suggest that even when people’s decisions are affected by many factors, the last option can come out on top.
